# Comparative Evaluation of Antibacterial Effect of Propolis and Aloe Vera, Xylitol, and Cpp-Acp Gels on *Streptococcus mutans* and *Lactobacillus* in Vitro

**DOI:** 10.1155/2021/5842600

**Published:** 2021-11-08

**Authors:** Maryam Hajiahmadi, Jamshid Faghri, Zohre Salehi, Fariba Heidari

**Affiliations:** ^1^Department of Pediatric Dentistry, Dental Research Center, School of Dentistry, Isfahan University of Medical Sciences, Isfahan, Iran; ^2^Department of Microbiology, School of Medicine, Isfahan University of Medical Sciences, Isfahan, Iran; ^3^Department of Pediatric Dentistry, School of Dentistry, Tehran University of Medical Sciences, Tehran, Iran; ^4^Torabinejad Dental Research Center, School of Dentistry, Isfahan University of Medical Sciences, Isfahan, Iran

## Abstract

**Introduction:**

Early childhood caries is a kind of caries occurring in deciduous teeth. Bacteria are among the main factors. Antibacterial agents such as fluoride are used in both prevention and treatment, but their application in children faces limitations such as fluorosis. Therefore, novel methods of caries prevention among the children are mainly focused on the use of fluoride-free active ingredients. In this comparative study, antibacterial effects of gels containing propolis and aloe vera, fluoride, xylitol, and CPP-ACP were investigated.

**Methods:**

This is an in vitro study. By plate well technique, plates containing gels were created in the culture medium of *Streptococcus mutans* and *Lactobacillus*, and their antibacterial impacts were evaluated by measuring the inhibition zone after 24, 48, and 72 hours. Then, different concentrations of each gel were evaluated in the same way for the antibacterial properties. For each sample, this process was iterated 3 times, where the average was declared as the final number. The collected data were entered in SPSS 24.

**Results:**

In both bacteria, propolis gel and aloe vera had the highest zone of inhibition, followed by fluoride and xylitol in the second and third places, respectively. Different concentrations of gels are significantly different in terms of antimicrobial effect (*P* value ≤ 0/05). The antimicrobial effect of propolis and aloe vera gel was kept up to the concentration of 1/16. As the bacterial and gel contact time is prolonged, the antibacterial effect of different gels increases, but the difference is not statistically significant (*P* value = 0.109). CPP-ACP gel had no antimicrobial effect at any concentration.

**Conclusion:**

Propolis and aloe vera gel had a greater antimicrobial effect than other gels, where such effect was observed in low concentrations. CPP-ACP gel had no antimicrobial properties.

## 1. Introduction

In recent years, awareness of the role the oral health plays in the quality of life of infants and young children, particularly their health and general well-being, has raised. On the other hand, World Health Organization (WHO) has stated that the prevalence of dental caries worldwide is increasing rapidly, and early childhood caries (ECC) is a serious health problem. ECC is an active and widespread caries in deciduous teeth that is currently often used to describe the dental caries among the young children [1, 2]. ECC can cause pain, tooth loss, and has negative effects on the quality of life. This early caries can also affect speech, appearance, and function and increases the risk of caries in the deciduous and permanent teeth [3]. ECC, like caries in the old age, is the result of the confrontation among the host, bacteria, oral environment, and passage of time; but this process is more complicated due to the age-related eating habits and anatomical features of the deciduous teeth [4, 5]. ECC, like other dental caries, is mainly affected by *Streptococcus mutans* and Lactobacilli. By fermenting carbohydrates, especially sucrose, and producing acid, these bacteria lead to demineralization of tooth enamel and consequently caries [6].

To prevent the tooth decay, various factors have been considered, among which fluoride is the golden standard. According to reports of MedlinePlus, fluoride therapy in the form of gel or mouthwash for preventing the tooth decay, acid resistance, and blocking of cavities made by bacteria is so useful [7, 8].

The literature and scientific discussions on the use and design of gel formulations for children have focused on the importance of fluoride dosage [9–11]. Fluoride intake with the age range of 15–30 months is of high importance, because at this age, excessive fluoride consumption can lead to fluorosis in the teeth, especially the anterior maxilla, which is aesthetically outstanding. This consumption can be worrying at younger or older ages, so it is necessary to replace the effective fluoride-free substance for ECC [12, 13].

Propolis can be found among these effective substances, which is a well-known natural antibiotic that affects caries by acting on the bacterial cell wall and inhibiting the bacterial motility [14]. Compounds in propolis inhibit the growth of *Streptococcus mutans* by inhibiting the activity of glucosyltransferase produced by *Streptococcus mutans*; the secreted ethanol has also an inhibitory impact on the growth of Lactobacillus [15, 16]. Airen et al. (2018) studied the antibacterial effect of propolis on *Streptococcus mutans* and Lactobacilli during an in vitro study and concluded that this substance is effective against both microorganisms [17].

Aloe vera is a plant from the Lilius family, whose gel is obtained from the leaves. Many studies have been conducted on the anti-inflammatory and antimicrobial properties of this plant [18]. Pharmacokinetic studies on aloe vera gel in vitro and in vivo have shown anti-inflammatory, antibacterial, and antioxidant properties. The phenol found in aloe vera can cause bacterial lysis, and its ethanol inhibits the growth of *Streptococcus mutans* and *Lactobacillus* [19, 20]. This feature can be used to resist bacteria as the main cause of caries. A study by Yetty Herdiyati Nonong et al. (2016), which compared the antimicrobial effect of aloe vera and sodium fluoride on *Streptococcus mutans*, revealed that aloe vera has the same antibacterial ability as the sodium fluoride due to the decreased number of *Streptococcus mutans* colonies [21].

Xylitol, as a good material for caries prevention, is a 5-carbon sugar made from plants and agricultural materials. Xylitol inhibits pathogen growth by selective antibacterial action against *Streptococcus mutans* and disrupts cell wall glucose transport and intracellular glycolysis [22]. It also reduces the adhesion of *Streptococcus mutans* to dental biofilms [23]. Xylitol inhibits the growth of *Streptococcus mutans* and *Lactobacillus* via fructose induction system and xylitol-5-phosphate formation [22, 24]. Xylitol-containing toothpaste reduces *S. mutans* colonies in saliva and increases saliva secretion as well as pH. These factors have positive effects on the quality of the oral medium, and inclusion of xylitol in preventive programs will be useful [25].

Swapnil oza et al. (2018) examined the effect of xylitol-containing chewing gum and sorbitol on *Streptococcus mutans* and Lactobacilli in saliva and plaque and found that only chewing gum containing xylitol influences the *Streptococcus mutans*, but both chewing gums were equally effective on Lactobacilli [26].

CPP-ACP is another effective caries prevention agent that acts as a reservoir of calcium and phosphate by combining nanocomplexes and plaque on the tooth surface. Studies show that CPP-ACP combined with dental plaque can significantly increase the plaque calcium and ion phosphate levels. This is an ideal mechanism to prevent the enamel demineralization, because there is a reverse relationship between calcium and phosphate on the plaque surface and caries [27]. CPP-ACP is also believed to have an antibacterial and buffering effect on plaque and interfere with the growth and adhesion of *Streptococcus mutans* species [28]. In a clinical study, Shweta Chandak et al. (2016) examined the impact of CPP-ACP alone and in combination with fluoride on the amount of *Streptococcus mutans* in children's dental plaque and concluded that both groups significantly reduced this microorganism, of which the rate is higher in the combination of these two substances [29].

## 2. Method and Material

This study was experimental, and its target population was *Streptococcus mutans* and *Lactobacillus*. Gels containing propolis and aloe vera (forever BRIGHT, manufactured by: Aloe vera of America, INC), CPP-ACP (GC Tooth Mousse, manufactured by GC America INC), xylitol (Nenedent, manufactured by Dentinox, Berlin, Germany), and fluoride 1000 PPM (Frice, manufactured by Seagull, Iran) were provided.

To evaluate the antimicrobial properties of gels in this study, standard strains of *Streptococcus mutans* (ATCC:35668) and *Lactobacillus acidophilus* (ATCC: 4356) were purchased from the Center for the Collection of Fungi and Industrial Bacteria of Iran. *Streptococcus mutans* and Lactobacilli were prepared according to the Half McFarland standard, which is equivalent to 1.5 × ^10^^^8^ CFU/ml of bacteria.

To prepare the half-McFarland turbidity, 3–4 colonies were removed from the initial 24-hour culture medium using a swap and suspended in a sterile Falcon tube containing sterile saline to create the turbidity of the half-McFarland tube.

To prepare the culture medium, 40 g of powder was poured into 950 milliliters of distilled water in Erlenmeyer and heated; Erlenmeyer was, then, placed in an autoclave for 15 minutes at 121°C to be sterilized. Once the solution reached a temperature of 45–50°C, 7% defibrillated blood was added to the solution for enrichment. The solution was then transferred to a 10 cm sterile plate and stored in the refrigerator.

50 Landa of the bacterial suspension equivalent to half of McFarland was cultured on the sterile medium by vacuum sterilization. After that, 6 wells with a diameter of 6 mm were created on each plate at regular intervals. In each plate, gels were injected into the wells in 100 Landa, and then the culture medium was incubated at 37°C and placed in the incubator. After 24, 48, and 72 hours, the inhibition zone around the gels was measured with a digital caliper (for each sample, this was iterated 3 times, and the average was declared as the final number).

Each plate had one positive control (culture medium alone) and one negative control (culture medium with bacteria).

In the following, to compare the antimicrobial activity of different concentrations of gels by plate well method, concentrations of 1/2, 1/4, 1/8, and 1/16 were prepared from each of the four existing toothpastes by serial dilution method. A series of sterile Falcon tubes was prepared. To obtain a concentration of 1/2, 1 cc of the desired toothpaste was mixed in 1 cc of distilled water, and a homogeneous liquid was obtained by a mix shaker. In the next step, by transferring 1 cc of this solution to 1 cc of distilled water, a concentration of 1/4 was achieved; the rest of the required concentrations were prepared in the same way.

Once the different concentrations were prepared, a culture suspension equivalent to half McFarland was prepared on blood agar culture medium by broom culture method with sterile swab culture and 6 wells (4 concentrations with positive and negative control) with a diameter of 6 mm on each plate at regular intervals; 100 Landa of different concentrations was placed in each well.

The culture medium was incubated at 37 °C, and after 24, 48, and 72 hours, the inhibition zone around the concentrations was measured with a digital caliper (for each example, this was iterated 3 times, and the average was declared as the final number).

Each plate had one positive control (culture medium alone) and one negative control (culture medium with bacteria).

## 3. Result

In studying the microbial inhibition zone, samples showed that:

1. By reducing the concentration, the antibacterial effect of different gels is decreased.
2. As the bacterial and gel contact time is prolonged, the antibacterial effect of different gels increases, but the difference is not statistically significant (*P* value = 0.109).
3. CPP-ACP gel has no antibacterial effect at any concentration.

Concerning the *Streptococcus mutans*, surveying the samples with digital caliper showed that (Table 1):

1. In all concentrations, propolis and aloe vera gel had an inhibitory effect on growth of *Streptococcus mutans*.
2. Fluoride gel up to a concentration of 1/8 had an inhibitory effect on growth of *Streptococcus mutans*.
3. Xylitol gel only in concentration 1 had an inhibitory effect on growth of *Streptococcus mutans*.

Also, studying the samples with concentration 1 after 72 hours showed the following:

1. The highest inhibition zone was associated to the gel containing propolis and aloe vera (10.81 ± 0.77 mm)
2. The second rank of inhibition zone was allocated to fluoride gel (7.74 ± 0.36 mm)
3. Xylitol gel (6.44 ± 0.41 mm) was ranked 3^rd^ as the lowest growth inhibition zone

These observations for *Lactobacillus* showed the following (Table 2):

1. Propolis and aloe vera gel, in all concentrations, had an inhibitory effect on growth of *Lactobacillus* bacteria
2. Fluoride gel, in all concentrations, had an inhibitory effect on growth of *Lactobacillus* bacteria
3. Xylitol gel up to a concentration of 1/2 had an inhibitory effect on growth of Lactobacilli
4. Propolis and aloe vera gel and fluoride gel in *Lactobacillus* bacteria had a larger growth inhibition zone than *Streptococcus mutans*

Investigating the samples with concentration 1 after 72 hours also showed the following:

1. The highest growth inhibition zone of this bacterium was related to propolis and aloe vera gel (14.26 ± 0.77 mm)
2. The second rank of inhibition zone was allocated to fluoride gel (11.57 ± 0.73 mm)
3. Xylitol gel (6.51 ± 0.32 mm) was ranked 3^rd^ as the lowest growth inhibition zone

### 3.1. *Streptococcus mutans*

Studying the growth inhibition zone in different concentrations of propolis and aloe vera gel by Kruskal–Wallis and Mann–Whitney test indicated that 5 different concentrations of propolis and aloe vera gel had a significant difference in antibacterial effect at all times (*P* value ≤ 0.05).

For fluoride gel, studying the diameter of the growth inhibition zone in different concentrations by Kruskal–Wallis test showed that there is a significant difference between various concentrations of fluoride gel at all times in terms of antibacterial effect (*P* value ≤ 0.05).

Comparing the results of different concentrations of the studied gels with each other at all times by Mann–Whitney test showed that:

Different concentrations of fluoride gel up to 1/4 concentration are significantly different in terms of antibacterial effect (*P* value ≤ 0.05). The antibacterial effect of concentrations 1/8 and 1/16 concentrations was not significant (*P* value = 0.317).

Regarding xylitol gel, comparing the diameter of the growth inhibition zone in different concentrations with Kruskal–Wallis test showed that there is a significant difference between various concentrations of xylitol gel in terms of antibacterial effect at all times (*P* value ≤ 0.05). Mann–Whitney test also showed that xylitol gel only at concentration 1 had an antibacterial effect on *Streptococcus mutans*, which was significantly different compared to the lower concentrations (*P* value ≤ 0.05).

Comparing the results of different concentrations of the studied gels with each other at all times by Mann–Whitney test showed the following:

1. There is a significant difference between the antibacterial effect of propolis and aloe vera gel and fluoride gel in concentrations 1, 1/2, 1/4, and 1/8 (*P* value  ≤  0.05). This difference was not significant at a concentration of 1/16 (*P* value = 0.317).
2. The antibacterial effect of propolis and aloe vera gel and xylitol gel is significantly different up to the concentration of 1/8 (*P* value≤ 0.05). This difference was not significant in concentration 1/16 (*P* value = 0.109).
3. There was a significant difference between the antibacterial effect of fluoride gel and xylitol gel only in concentrations of 1, 1/2, and 1/4 (*P* value ≤ 0.05).

This difference was not significant in the concentrations 1/8 (*P* value = 0.317) and 1/16 (*P* value = 1.000).

### 3.2. *Lactobacillus*

Investigating the diameter of the growth inhibition halo in different concentrations of propolis and aloe vera gel by Kruskal–Wallis test revealed that there is a significant difference between 5 different concentrations of propolis and aloe vera gel in terms of antibacterial effect (*P* value ≤ 0.05). Also, this analysis with Mann–Whitney test showed that this difference was significant between concentrations 1/2 and 1/4 and between 1/4 and 1/8 concentrations after 72 and 48 hours, respectively (*P* value ≤ 0.05).

For fluoride gel, studying the diameter of the growth inhibition zone in different concentrations by Kruskal–Wallis and Mann–Whitney tests showed that there is a significant difference between different concentrations of fluoride gel in all three times in terms of antibacterial effect (*P* value ≤ 0.05).

Regarding xylitol gel, comparing the diameter of growth inhibition zone in different concentrations with Kruskal–Wallis test showed that there is a significant difference between different concentrations of xylitol gel in terms of antibacterial effect in all three times (*P* value ≤ 0.05). Mann–Whitney test also indicated that the xylitol gel has an antibacterial effect on *Lactobacillus* only in concentrations 1 and 1/2 as it was significant compared to lower concentrations (*P* value ≤ 0.05).

Comparing the results of different concentrations of the studied gels with each other at all times by Mann–Whitney test showed the following:

1. There is a significant difference between the antibacterial effect of propolis and aloe vera gel and fluoride gel in all concentrations (*P* value ≤ 0.05).
2. The antibacterial effects of propolis and aloe vera gel and xylitol gel are significantly different in all concentrations (*P* value ≤ 0.05).

There is a significant difference between the antibacterial effect of fluoride gel and xylitol gel up to the concentration 1/8 (*P* value≤ 0.05). This difference was not significant at concentration 1/16 (*P* value = 0.317).

## 4. Discussion

In this study, a gel containing propolis and aloe vera had an antibacterial effect on *Streptococcus mutans* and Lactobacilli in all concentrations, even in concentration 1/16.

EA Ophori et al. (2010) [30], who studied the diameter of the growth inhibition zone of *Streptococcus mutans* by ethanol extracted from propolis, concluded that the propolis has the antibacterial properties in different concentrations (2 to 32 *μ*g/ml), which is consistent with our study.

The antimicrobial activity of propolis is widely supported by evidence [31]. Some studies have found that the propolis samples are only active against the Gram-positive bacteria and some fungi [32]. However, others have confirmed its activity against the Gram-negative bacteria [33]. Sforcin et al. [33] confirmed that growth of Gram-positive bacteria was inhibited by low concentrations of propolis (0.4%). In 2013, a study by Arkadiusz Dziedzic et al. [15] showed the antibacterial impact of propolis on *Streptococcus mutans* and Lactobacilli collected from saliva.

Fani and Kohanteb (2012) [34] concluded that well-concentrated aloe vera gel could be used as an antiseptic to prevent the tooth decay and periodontal disease.

In an empirical study by Karuna Yarmunja Mahabala et al. (2016) [20], ethanol was isolated from propolis and aloe vera leaves, and its antibacterial properties were examined. It was found that both of these substances have the antibacterial properties and this feature in aloe vera up to the concentration 1/4 and propolis at the concentration 1 on *Streptococcus mutans* and both are present only at the concentration 1 on *Lactobacillus*, which does not agree with the results of this study. Such difference in results may be due to the different shapes of the consumable and the additives in the gel in both studies.

The present study showed that there is a significant difference between the antimicrobial effect of propolis and aloe vera gel and fluoride gel in both bacteria, which is maintained up to 1/8 and 1/16 for *Streptococcus mutans* and *Lactobacillus*, respectively.

This result was consistent with the study by Fereyduni (2013) [35] that compared the effect of propolis toothpaste with regular toothpaste (containing fluoride) on the microbial plaque. Results of this study are also consistent with the studies by Morawiec et al. [36], Tanasiewicz et al. [37], and Skaba et al. [38].

A study by Dilip George et al. (2009) [39] showed the antimicrobial effect of aloe vera gel and two fluoride-containing toothpastes (Pepsodent and Colgate) on various bacterial strains including *Streptococcus mutans* and *Lactobacillus acidophilus*. This study was consistent with a study by Yetty Herdiyati Nonong et al. in 2016 [21] that compared the antimicrobial effects of aloe vera and sodium fluoride on *Streptococcus* mutans and found that aloe vera has the same antibacterial ability as sodium fluoride.

A study by Bertolini et al. (2012) [40] on the effect of propolis and aloe vera gel on *Streptococcus mutans* species in toothbrushes concluded that there was no significant difference between the antimicrobial effect of propolis and aloe vera gel and other antimicrobials including fluoride. Probable causes of this discrepancy include the use of higher concentrations of fluoride (PPM1450) in Bertolini's study.

Results of this study showed that the antimicrobial effect of propolis and aloe vera gel was significantly higher than xylitol gel in both bacteria, and this difference was kept for the *Streptococcus mutans* up to 1/8 and *Lactobacillus* up to 1/16. This result was consistent with a study by Sneha Girdhari Tulsani et al. (2014) [41].

In this study, it was observed that the gel containing xylitol has an antibacterial impact on both *Streptococcus mutans* and *Lactobacillus*, and in the case of Lactobacillus, this effect was also present in the concentration 1/2. This result was in line with the studies of Hamim Fithrony 2009 [42], zhan *l* 2012 [43].

In a study by S. Radmerikhi et al. (2013) [44], a significant difference was found between the antibacterial properties of different concentrations of xylitol (18%, 12%, 8%, and 3%) on both *Streptococcus mutans* and *Lactobacillus* and all of these concentrations have the antibacterial effects. Compared to the antibacterial properties of different concentrations of studies, such difference can be made due to the amount of different active ingredients of each substance depending on the form of consumption, manufacturer, differences in laboratory methods, and type of data analysis. Also, this effect on *Lactobacillus* is higher than that on *Streptococcus mutans*, which is similar to the present study.

Results of our study include more antibacterial properties of fluoride-containing gels on both bacteria than xylitol gel. In a systematic review study by Yu Wang et al. (2017) [45], they investigated the impact of nonfluoridated agents such as chlorhexidine, arginine, triclosan, xylitol, and CPP-ACP on the deciduous teeth compared with placebo or fluoride. Xylitol wipes were effective in controlling tooth decay in primary teeth. However, a study evaluating the products containing low doses of xylitol (0.5–1.5 g/tablet) found no cariostatic activity of xylitol.

Results of this study showed that CPP-ACP has no antibacterial effect at any concentration.

In their study, Ruchi Vashisht et al. (2013) [28] investigated the effect of CPP-ACP on remineralization of lesions and growth inhibition of *Streptococcus mutans* and concluded that *S. mutans* decreased in the intervention group 3 months after evaluation. CPP-ACP also has the antibacterial and buffering impacts on the plaque and interferes with growth and adhesion of *Streptococcus* species, but they reported that a decrease in *S. mutans* could not be attributed to the inhibitory effect of CPP-ACP alone, because there is a significant reduction in the control group, and these reductions in both groups may be associated to the changes in children's oral health behaviors, since all children at the beginning of the study met the oral health education more and consume less cariogenic product than before.

In an in vitro study by Erdem et al. (2011) [46], it was reported that, after using CPP-ACP, the survival of *S. mutans* in biofilm decreased, but this decrease was not statistically significant.

Limited studies have been conducted on *Lactobacillus* bacteria containing CPP-ACP, and most studies have focused on *Streptococcus mutans*.

In recent studies that focused on the effect of CPP-ACP products on bacterial counting, acidogenicity, and relative abundance of bacteria species associated with caries and health, it was observed that CPP-ACP can change bacterial biofilm environment to nonpathogenic bacteria and weaken bacterial virulence [47–49].

According to our results, gels containing propolis and aloe vera have more antibacterial properties than fluoride gel (PPM1000) and seemingly, it can be used with less concern among the young children at risk of swallowing too much fluoride. The result is effective in preventing early childhood caries. Higher concentrations of fluoride may be more bacteriostatic, but this advantage must be weighed against the likelihood of fluorosis.

In general, propolis is safe and nontoxic and does not irritate most people when used. However, like other bee products, there are people who are allergic to them [50]. Aloe vera should also be used with caution in people with a history of allergies to plants from the Liliaceae family [51]. Therefore, more research is needed on the side effects of topical application of propolis and aloe vera gel.

## 5. Conclusion

Propolis and aloe vera, fluoride, and xylitol gels have an inhibitory effect on growth of *Streptococcus mutans* and *Lactobacillus*. CPP-ACP gel has no experimentally antimicrobial activity.

Propolis and aloe vera gel have a greater antibacterial effect than other gels, which keeps this property even in lower concentrations.

## Figures and Tables

**Table 1:** Mean diameter ± standard deviation of *Streptococcus mutans* growth inhibition zone in different concentrations of each gel.

Concentration 1/16	Concentration 1/8	Concentration 1/4	Concentration 1/2	Concentration 1	Time (h)	Groups	
0.23 ± 0.40	2.04 ± 0.07	4.22 ± 0.34	5.12 ± 0.18	7.74 ± 0.51	24	Propolis and aloe vera gel	1
0.33 ± 0.57	2.49 ± 0.24	5.02 ± 0.72	6.47 ± 0.73	8.85 ± 1.03	48		
0.43 ± 0.75	2.99 ± 0.26	5.57 ± 0.79	7.72 ± 0.92	10.81 ± 0.7	72		

0	0.30 ± 0.51	1.45 ± 0.36	2.65 ± 0.31	6.12 ± 0.15	24	Fluoride gel	2
0	0.33 ± 0.57	1.91 ± 0.47	3.18 ± 0.37	7.02 ± 0.53	48		
0	0.40 ± 0.69	2.05 ± 0.52	3.31 ± 0.33	7.74 ± 0.36	72		

0	0	0	0	5.84 ± 0.07	24	Xylitol gel	3
0	0	0	0	6.07 ± 0.06	48		
0	0	0	0	6.44 ± 0.41	72		

0	0	0	0	0	24	CPP-ACP gel	4
0	0	0	0	0	48		
0	0	0	0	0	72		

**Table 2:** Mean diameter ± standard deviation of *Lactobacillus* growth inhibition zone in different concentrations of each gel.

Concentration 1/16	Concentration 1/8	Concentration 1/4	Concentration 1/2	Concentration 1	Time (h)	Groups	
2.57 ± 0.60	5.54 ± 0.66	6.24 ± 0.59	6.96 ± 0.51	8.05 ± 0.27	24	Propolis and aloe vera gel	1
4.44 ± 0.67	5.82 ± 0.39	7.09 ± 0.61	8.11 ± 0.60	11.50 ± 0.74	48		
5.51 ± 0.56	7.16 ± 0.33	8.99 ± 0.28	10.61 ± 0.4	14.26 ± 0.77	72		

0	2.19 ± 0.85	3.97 ± 0.77	5.13 ± 0.22	6.43 ± 0.30	24	Fluoride gel	2
0.33 ± 0.57	3.11 ± 0.83	4.92 ± 0.98	6.25 ± 0.21	8.85 ± 0.15	48		
0.85 ± 0.87	4.55 ± 0.78	6.27 ± 0.25	7.66 ± 0.29	11.57 ± 0.73	72		

0	0	0	2.21 ± 0.54	4.01 ± 0.05	24	Xylitol gel	3
0	0	0	2.94 ± 0.15	4.71 ± 0.23	48		
0	0	0	4.07 ± 0.15	6.51 ± 0.32	72		

0	0	0	0	0	24	CPP-ACP gel	4
0	0	0	0	0	48		
0	0	0	0	0	72		

## Data Availability

The data are available from the corresponding author upon reasonable request.
